# Supplementary material for: Quantitative dissection of transcription in development yields evidence for transcription-factor-driven chromatin accessibility
Source: eLife. 2020 Oct 19;9:e56429. doi: 10.7554/eLife.56429 (PMC7738189; doi:10.7554/eLife.56429)
Supplement: Source code 1. [file elife-56429-code1.zip › src/mcmcstat-master/docs/ex/algaefun.html]

algaefun 

```
function y=algaefun(time,theta,y0,xdata)
% algae model function

[t,y] = ode15s(@algaesys,time,y0,[],theta,xdata);
```

Published with MATLAB® R2018b
