# Supplementary material for: Quantitative dissection of transcription in development yields evidence for transcription-factor-driven chromatin accessibility
Source: eLife. 2020 Oct 19;9:e56429. doi: 10.7554/eLife.56429 (PMC7738189; doi:10.7554/eLife.56429)
Supplement: Source code 1. [file elife-56429-code1.zip › src/mcmcstat-master/docs/ex/algaess.html]

algaess 

```
function ss = algaess(theta,data)
% algae sum-of-squares function

time   = data.ydata(:,1);
ydata  = data.ydata(:,2:end);
xdata  = data.xdata;

% 3 last parameters are the initial states
y0 = theta(end-2:end);

ymodel = algaefun(time,theta,y0,xdata);
ss = sum((ymodel - ydata).^2);
```

Published with MATLAB® R2018b
