# Supplementary material for: Quantitative dissection of transcription in development yields evidence for transcription-factor-driven chromatin accessibility
Source: eLife. 2020 Oct 19;9:e56429. doi: 10.7554/eLife.56429 (PMC7738189; doi:10.7554/eLife.56429)
Supplement: Source code 1. [file elife-56429-code1.zip › src/mcmcstat-master/docs/ex/cauchyex.html]

cauchyex 

MCMC toolbox » Examples » Cauchy distribution

## MCMC toolbox example

This example targets 10 dimensional Cauchy distribution. Cauchy distribution does not have finite second moment, so the AM method, which calculates chain variance will have troubles. In practice, it gives good results. Here, we use RAM adaptation, which does not use variance information (http://dx.doi.org/10.1007/s11222-011-9269-5).

```
clear model options params

nsimu = 40000;
npar = 10;

model.ssfun    = @(x,d) 2*sum(log(1+x.^2));
options.nsimu  = nsimu;
options.method = 'ram';
for i=1:npar, params{i} = {sprintf('x_{%d}',i), 0}; end

[res,chain] = mcmcrun(model,[],params,options);
```

```
Sampling these parameters:
name   start [min,max] N(mu,s^2)
x_{1}: 0 [-Inf,Inf] N(0,Inf)
x_{2}: 0 [-Inf,Inf] N(0,Inf)
x_{3}: 0 [-Inf,Inf] N(0,Inf)
x_{4}: 0 [-Inf,Inf] N(0,Inf)
x_{5}: 0 [-Inf,Inf] N(0,Inf)
x_{6}: 0 [-Inf,Inf] N(0,Inf)
x_{7}: 0 [-Inf,Inf] N(0,Inf)
x_{8}: 0 [-Inf,Inf] N(0,Inf)
x_{9}: 0 [-Inf,Inf] N(0,Inf)
x_{10}: 0 [-Inf,Inf] N(0,Inf)
```

## Plot the chain

```
iii = 1:min(npar,4); % plot first 4 chain columns

figure(1); clf
mcmcplot(chain,iii,res);
figure(2); clf
mcmcplot(chain,iii,res,'hist');
for i=iii
  subplot(2,2,i)
  xlim([-10,10]);
  xx = linspace(-10,10);
  yy = cauchypf(xx);
  hold on
  plot(xx,yy)
  hold off
end
```

 

Published with MATLAB® R2018b
