# Supplementary material for: Quantitative dissection of transcription in development yields evidence for transcription-factor-driven chromatin accessibility
Source: eLife. 2020 Oct 19;9:e56429. doi: 10.7554/eLife.56429 (PMC7738189; doi:10.7554/eLife.56429)
Supplement: Source code 1. [file elife-56429-code1.zip › src/mcmcstat-master/docs/ex/himmelode.html]

himmelode 

```
function ydot = himmelode(t,y,k)
% Himmelblau 9.9 odefile
A=y(1); B=y(2); C=y(3); D=y(4);
ydot = [
    -k(1)*A*B - k(2)*A*C - k(3)*A*D;
    -k(1)*A*B;
    +k(1)*A*B - k(2)*A*C;
              + k(2)*A*C - k(3)*A*D;
                         + k(3)*A*D;
       ];
```

Published with MATLAB® R2018b
