# Supplementary material for: Quantitative dissection of transcription in development yields evidence for transcription-factor-driven chromatin accessibility
Source: eLife. 2020 Oct 19;9:e56429. doi: 10.7554/eLife.56429 (PMC7738189; doi:10.7554/eLife.56429)
Supplement: Source code 1. [file elife-56429-code1.zip › src/mcmcstat-master/docs/ex/himmelss.html]

himmelss 

```
function ss = himmelss(k,data)
% sum-of-squares for Himmelblau 9.9

time = data.ydata(:,1);
Aobs = data.ydata(:,2);
y0   = data.y0;

[t,y] = ode45(@himmelode,time,y0,[],k);
Amodel = y(:,1);

ss = sum((Aobs-Amodel).^2);
```

Published with MATLAB® R2018b
